# Blockchain for Securing AI-Driven Healthcare Systems: A Systematic Review and Future Research Perspectives

**DOI:** 10.7759/cureus.83136

**Published:** 2025-04-28

**Authors:** Pratik Kasralikar, Omkar Reddy Polu, Balaiah Chamarthi, Rana Veer Samara Sihman Bharattej Rupavath, Sandipkumar Patel, Ramakrishna Tumati

**Affiliations:** 1 Department of Business Administration, Lindsey Wilson College, Kentucky, USA; 2 Department of Technology and Innovation, City National Bank, Los Angeles, USA; 3 Department of Technology and Innovation, Info Services LLC, Livonia, USA; 4 Department of Business Administration, National Louis University, Tampa, USA; 5 Department of Computer Engineering, Gujarat Technological University, Ahmedabad, IND; 6 Department of Software Application and Engineering, Intel, Beaverton, USA

**Keywords:** artificial intelligence (ai), blockchain, data privacy, federated learning, healthcare security

## Abstract

The integration of AI in healthcare has significantly advanced diagnostics, patient monitoring, and personalized treatments. However, the reliance on vast datasets raises critical concerns about data privacy, security, and trustworthiness. Blockchain technology, with its decentralized and immutable nature, has emerged as a promising solution to these challenges. This systematic review aims to explore the role of blockchain in securing AI-driven healthcare systems, evaluating its potential to enhance data security, privacy, and interoperability while identifying key challenges and future research directions. The review followed the Preferred Reporting Items for Systematic Reviews and Meta-Analyses (PRISMA) guidelines, conducting a comprehensive search across databases such as IEEE (Institute of Electrical and Electronics Engineers) Xplore Digital Library, Scopus, Web of Science, and PubMed. Inclusion criteria focused on peer-reviewed studies discussing blockchain and AI integration in healthcare, while exclusion criteria eliminated irrelevant or non-empirical studies. Data extraction captured study characteristics, AI algorithms, blockchain types, key findings, and limitations. Quality assessment was performed using the Joanna Briggs Institute (JBI) Critical Appraisal Checklist, evaluating methodological rigor, innovation, and reproducibility. The review included 15 studies highlighting blockchain's role in securing AI-driven healthcare systems. Key findings demonstrated blockchain's effectiveness in enabling decentralized data sharing (e.g., federated learning (FL)), enhancing data integrity, and improving diagnostic accuracy. However, challenges such as scalability, interoperability, and regulatory compliance were recurrent. The quality assessment revealed moderate-to-high methodological quality but underscored gaps in reproducibility and real-world validation. Blockchain technology holds transformative potential for securing AI-driven healthcare systems by addressing critical privacy and security concerns. However, the field remains nascent, with significant hurdles in scalability, technical standardization, and ethical governance. Future research should prioritize hybrid blockchain architectures, clinical trials, and interdisciplinary collaboration to bridge these gaps and realize the full potential of blockchain-AI integration in healthcare.

## Introduction and background

The use of advanced technologies in healthcare is rapidly transforming the field, and one of the most significant developments is the integration of AI. The integration of AI in healthcare has revolutionized various aspects of medical practice, including diagnostics, patient monitoring, and personalized treatments [1]. AI refers to computer systems that can perform tasks typically requiring human intelligence, such as analyzing medical images or predicting health outcomes. AI-powered healthcare systems, which rely heavily on machine learning algorithms and vast datasets, offer significant improvements in accuracy, efficiency, and accessibility [2]. These advancements have the potential to greatly enhance patient care, reduce healthcare costs, and streamline the decision-making process. However, the widespread adoption of AI in healthcare introduces new challenges, primarily centered on data privacy, security, and trustworthiness [3]. AI systems require large volumes of medical data, which can include sensitive patient information such as medical histories, genetic profiles, and imaging data. Machine learning models improve over time by learning from such data, but this also increases the risk of misuse if the data is not well protected. Given the sensitive nature of this data, ensuring its privacy and security is paramount, as any breach or misuse could have serious ethical, legal, and financial implications [4].

One of the most pressing concerns in AI-driven healthcare systems is the vulnerability to cyberattacks and data breaches. Traditional centralized systems, where data is stored in a single location or managed by a central authority, are susceptible to single points of failure, making them prime targets for hackers [5]. Moreover, the sharing of medical data across institutions, often necessary for collaborative research and AI model training, exacerbates these concerns, as it introduces additional vectors for potential data misuse. This is especially problematic when institutions use different systems that may not be equally secure. Furthermore, existing methods for securing healthcare data, such as encryption and access controls, may not be sufficient to address the complex challenges posed by the vast, decentralized, and dynamic nature of healthcare data [6]. Encryption helps protect data by turning it into unreadable code, and access controls limit who can view or change the data, but these methods alone are not always enough in large, complex healthcare systems. These concerns underscore the importance of developing more robust and innovative solutions for safeguarding patient information while enabling the full potential of AI in healthcare.

In recent years, Blockchain technology has emerged as a promising solution to these security and privacy challenges [7]. Blockchain is a decentralized, distributed ledger technology that records transactions across multiple computers in a way that ensures the security, transparency, and immutability of data. In simpler terms, Blockchain works like a digital record book shared across many users, where each change is recorded and cannot be easily altered. Its inherent characteristics, such as decentralization, cryptographic security, and consensus mechanisms, make it an ideal candidate for securing AI-driven healthcare systems [8]. Blockchain provides an immutable record of all data transactions, making it possible to trace the origin of data, verify its integrity, and ensure transparency in its use. Furthermore, blockchain enables the creation of decentralized applications (DApps) that allow for secure data sharing and collaboration between institutions without the need for a central authority, thereby addressing the concerns of privacy and security [9]. These applications can function across hospitals and research centers, promoting secure collaboration while reducing the risk of a single point of failure.

Despite the potential of blockchain to revolutionize the security landscape of AI-driven healthcare systems, there are still significant hurdles to overcome in its implementation. These include challenges related to scalability, interoperability with existing healthcare infrastructure, regulatory compliance, and the energy consumption associated with blockchain networks [10]. For example, integrating blockchain with older hospital systems can be complex, and the high energy demands of some blockchain platforms raise concerns about sustainability. Moreover, while blockchain can enhance data security, it is not a panacea, and its integration with AI systems requires careful consideration of various technical, ethical, and legal factors.

The primary objective of this systematic review is to explore the intersection of blockchain and AI in healthcare, with a focus on how blockchain can be utilized to secure AI-driven healthcare systems. Through a comprehensive analysis of the existing literature, this review aims to evaluate the current state of research on blockchain-based solutions for healthcare data security, identify the key challenges and limitations, and highlight the potential benefits and applications of these technologies in clinical and administrative healthcare settings. By synthesizing the findings from various studies, this review seeks to provide valuable insights into the current progress and gaps in this rapidly evolving field and offer directions for future research.

## Review

Methodology

Review Protocol

The review was conducted in alignment with established guidelines for systematic reviews, particularly the PRISMA (Preferred Reporting Items for Systematic Reviews and Meta-Analyses) statement [11], ensuring transparency, reproducibility, and methodological rigor.

The primary objective of this review was to assess the role of blockchain technology in securing AI-driven healthcare systems. The focus was on evaluating areas such as data privacy, data integrity, transparency, security, and the ethical issues related to the integration of AI and blockchain in healthcare systems. This review seeks to provide a comprehensive analysis of the potential benefits and challenges of integrating blockchain technology into AI-driven healthcare solutions.

Inclusion and Exclusion Criteria

To ensure the reliability and relevance of the studies included, strict inclusion and exclusion criteria were defined.

The inclusion criteria for this review were as follows: only peer-reviewed journal articles, conference proceedings, white papers, and preprints were considered. The studies included had to explicitly discuss AI applications in healthcare, particularly those integrating blockchain technology to enhance data security, privacy, and interoperability. Relevant study types included empirical studies, conceptual papers, case studies, technical papers, and systematic reviews. The review focused on studies addressing data privacy, integrity, decentralized data sharing, AI model security, and ethical concerns related to blockchain and AI integration in healthcare systems.

The exclusion criteria for this review were as follows: studies that do not focus on AI-driven healthcare or the integration of blockchain technology were excluded. Additionally, studies lacking empirical data or models related to the research questions were not considered. Articles that focused on healthcare technologies unrelated to AI or blockchain, as well as theoretical papers or studies that only mentioned blockchain without addressing AI applications, were also excluded.

Search Strategy

A comprehensive search strategy was developed to ensure a broad and inclusive selection of studies. The following electronic databases were queried: IEEE (Institute of Electrical and Electronics Engineers) Xplore, PubMed, ScienceDirect, ACM Digital Library, Google Scholar, and SpringerLink. Search terms included combinations of keywords such as “Blockchain technology,” “AI in healthcare,” “Data security in healthcare,” “Privacy and Blockchain in healthcare,” “Federated learning,” and “Decentralized healthcare systems.” No restrictions were placed on the type of publication to ensure comprehensive coverage, and reference lists from the included studies were also examined for additional relevant studies.

Study Selection

The study selection process involved two stages: screening of titles and abstracts followed by full-text assessments. In the first stage, an initial review of titles and abstracts was conducted to eliminate irrelevant articles. Full-text versions of potentially eligible studies were then assessed for inclusion according to the pre-defined criteria.

Two independent reviewers (ORP and BC) from the list of authors with expertise in blockchain, AI, and healthcare systems performed the screening. Any discrepancies in the selection process were resolved through discussion, and a third reviewer (SP) was consulted when necessary. Only studies that met the inclusion criteria were included for further analysis.

Data Extraction

Data extraction was carried out systematically to capture key information from each included study. An extraction form was designed to collect relevant data to inform the synthesis of findings. The following details were extracted from each study: study information such as author(s), publication year, title, journal or conference proceedings, and study design; study focus, specifically identifying AI applications in healthcare (e.g., diagnostics, patient monitoring, decision support); blockchain integration, including a detailed description of how blockchain was utilized (e.g., smart contracts, federated learning (FL), decentralized networks) to secure AI-driven healthcare systems; key findings related to data privacy, data security, transparency, trust, and system performance; challenges identified, including issues related to scalability, regulatory concerns, and system interoperability in integrating blockchain with AI; and conclusions and future research directions, focusing on insights provided by the authors about the integration of blockchain and AI in healthcare and suggested areas for future research. Two reviewers independently performed the data extraction, and disagreements were resolved through discussion.

Quality Assessment

The quality assessment was conducted using the JBI (Joanna Briggs Institute) Critical Appraisal Checklist for Quasi-Experimental Studies, adapted to evaluate technical research on blockchain and AI-driven healthcare systems. This tool was selected for its structured approach to assessing methodological rigor, clarity, and validity in non-randomized studies. Six criteria were adopted: (1) clarity of research objectives, (2) detailed description of AI algorithms and blockchain architecture, (3) use of quantitative evaluation metrics, (4) discussion of limitations/threats to validity, (5) reproducibility (availability of code/datasets), and (6) innovation/impact. Each criterion was scored as yes (2), partial (1), or no (0), with a maximum total score of 12. Two independent reviewers performed the assessments to ensure consistency, with discrepancies resolved through consensus.

Data Synthesis

A narrative synthesis approach was used to analyze and structure the findings from the selected studies. The extracted data were categorized into key thematic areas to better understand the role of blockchain in securing AI-driven healthcare systems. These categories included the types of blockchain technologies employed (such as public, private, or consortium blockchains), the specific security mechanisms provided by blockchain for enhancing data privacy, security, and integrity in healthcare, and the integration methods used to combine AI and blockchain (e.g., smart contracts, FL). Additionally, the synthesis captured the reported benefits of this integration, such as secure model training and decentralized data sharing, as well as the challenges like scalability issues and regulatory barriers that need to be addressed for broader adoption.

Results

Studies Selection Process

**Figure 1:**
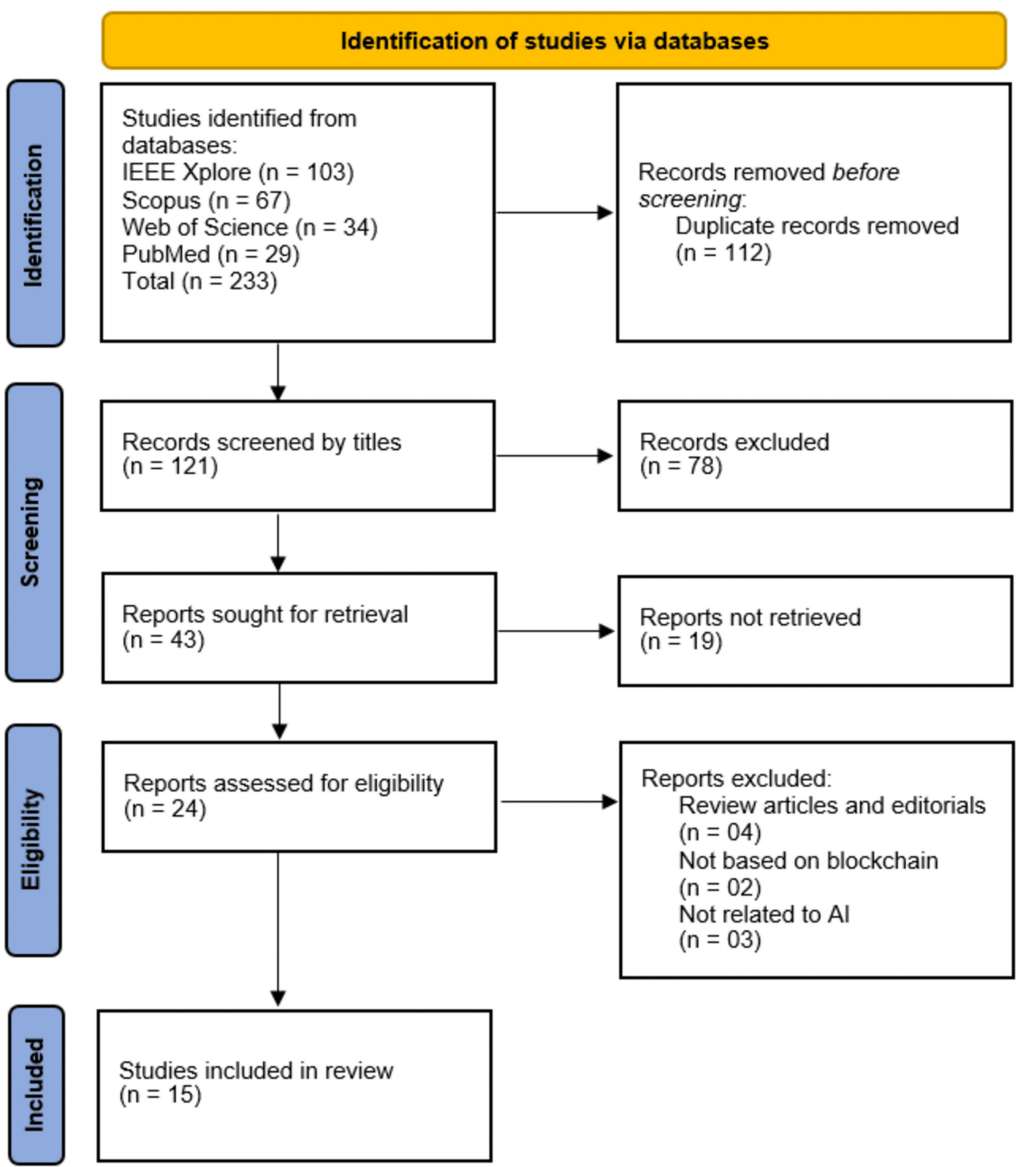
PRISMA Flowchart of Study Selection Process PRISMA, Preferred Reporting Items for Systematic Reviews and Meta-Analyses; IEEE, Institute of Electrical and Electronics Engineers

The systematic search across four databases, IEEE Xplore (n = 103), Scopus (n = 67), Web of Science (n = 34), and PubMed (n = 29), yielded 233 initial records. After removing 112 duplicate records, 121 unique studies underwent title and abstract screening. Of these, 78 records were excluded due to irrelevance to the research scope, leaving 43 reports sought for full-text retrieval. However, 19 articles could not be accessed or retrieved, resulting in 24 full-text publications assessed for eligibility. During eligibility screening, nine studies were excluded: four review articles/editorials, two studies unrelated to blockchain, and three studies not incorporating AI. Ultimately, 15 studies met the inclusion criteria and were incorporated into the systematic review (Figure 1).

Characteristics of Included Studies

The systematic review included 15 studies [12-26] published between 2017 and 2024, focusing on the integration of blockchain and AI to enhance security, privacy, and decentralization in healthcare systems. A thematic analysis revealed three dominant objectives: (1) improving data security and privacy (e.g., FL frameworks for medical Internet of Things (IoT) devices by Połap et al. [13] and Rahman et al. [15]), (2) enabling decentralized AI model training and data sharing (e.g., Zerka et al.’s [14] C-DistriM platform for multicentric medical imaging), and (3) addressing domain-specific challenges such as disease diagnosis (e.g., Kumar et al.’s [21] lung cancer detection using CT scans) and stigma reduction (e.g., Pilozzi & Huang’s [16] NLP-blockchain system for Alzheimer’s disease).

The studies employed diverse AI algorithms, including deep learning (Mamoshina et al. [12], Tan et al. [22]), federated/distributed learning (Zerka et al. [14], Kumar et al. [23]), and neural networks (Kim & Huh [17], Khan et al. [26]). Blockchain implementations varied across decentralized (Mamoshina et al. [12]), permissioned (Kuo et al. [20]), and public architectures (Puri et al. [25]), with hybrid approaches integrating smart contracts (Rahman et al. [15]) or InterPlanetary File System (IPFS) for secure storage (Gupta et al. [18]). Key innovations included enhanced data confidentiality (e.g., Kim & Huh’s [17] personal health record (PHR) verification framework), transparency in distributed AI training (Zerka et al. [14]), and blockchain-managed FL (Rahman et al. [15]).

Notably, medical applications dominated the corpus, with studies targeting disease detection (e.g., COVID-19 via CT scans by Kumar et al. [21]), retinal diagnostics (Tan et al. [22]), and anomaly recognition in stomach imaging (Khan et al. [26]). However, limitations persisted, including technical challenges such as unresolved scalability issues (Kuo et al. [20]), underexplained blockchain architectures (Jennath et al. [19]), and inadequate validation for real-world adoption (Pilozzi & Huang [16]). Only five studies explicitly addressed these limitations in depth, while others omitted critical discussions (e.g., Nguyen et al. [24]; Puri et al. [25]). Collectively, these studies highlight the potential of blockchain-AI integration but underscore the need for standardized frameworks and rigorous validation in healthcare contexts (Table 1).

**Table 1:** Summary of All Included Studies AD: Alzheimer’s disease, AUC: area under the curve, BITS: Blockchain-Based Intelligent Telesurgery System, C-DistriM: blockchain for privacy-preserving and trustworthy distributed machine learning in multicentric medical imaging, CNN: convolutional neural network, DBN: deep belief network, DL: deep learning, EHR: electronic health record, FL: federated learning, GDPR: General Data Protection Regulation, HIPAA: Health Insurance Portability and Accountability Act, IoHT: Internet of Health Things, IoT: Internet of Things, IPFS: InterPlanetary File System, NLP: natural language processing, PHR: Personal Health Record, RCNN: recurrent convolutional neural network, ResNet: residual network, SNARKs: succinct non-interactive argument of knowledge, TS: telesurgery

Author	Publishing Year	Objective	AI Algorithm	Type of Blockchain	Key Findings	Limitations
Mamoshina et al. [12]	2017	To propose a distributed, safe, and blockchain-based marketplace for DL technology in the healthcare sector.	Deep learning and transfer learning	Decentralized, secure, and transparent distributed blockchain	Deep learning and blockchain can empower patients to control and benefit from their health data while advancing biomedical research and healthcare innovation.	Data leaking is not prevented by the suggested solution after it is sold.
Połap et al. [13]	2020	Blockchain technology for the Internet of Medical Things' security and privacy.	FL (with neural networks)	Blockchain-based security (specific type not mentioned)	Decentralized FL with blockchain can enhance secure, privacy-preserving AI-assisted medical diagnosis and remote patient monitoring.	FL does not provide a suitable blockchain architecture.
Zerka et al. [14]	2020	To encourage transparency and confidence in research that is multicentric and ultimately to accelerate the implementation of AI.	C-DistriM, compared with centralized learning; uses predictive modeling (AUC comparison)	Blockchain-based platform integrated with sequential C-DistriM	The C-DistriM model provides equivalent performance to centralized models while enhancing transparency, trust, and feasibility in distributed AI training.	To investigate the behavior of C-DistriM in the event where dishonest collaborators are purposefully added to the network.
Rahman et al. [15]	2020	To safeguard the security and privacy of data from the IoHT.	FL, LD	Blockchain with smart contracts and encryption (specific type not mentioned)	The proposed blockchain-managed FL framework enhances privacy, trust, and security in IoHT-based healthcare using encrypted, decentralized training with strong adoption potential.	Fully decentralized FL remains a challenge due to limited training capability at nodes, scarcity of high-quality data, and complex authentication requirements.
Pilozzi and Huang [16]	2020	To examine the ways in which blockchain and AI can combat the stigma associated with Alzheimer's disease.	NLP for sentiment and tone analysis	Decentralized blockchain-based data storage and transfer (type not specified)	NLP can help assess public stigma toward AD, and blockchain may alleviate data privacy concerns and empower patients.	Implementation is theoretical; real-world adoption and institutional trust issues remain unaddressed.
Kim and Huh [17]	2020	To use blockchain algorithms powered by AI to offer safe PHR data verification and precise medical record verification in healthcare facilities.	Neural networks (mentioned as part of AI integration)	Information security blockchain system (specific type not detailed)	The proposed framework enhances data confidentiality, and extraction accuracy and supports standardization for secure healthcare data sharing.	Current systems like EHR are underutilized due to unresolved security and interoperability challenges.
Gupta et al. [18]	2020	To address concerns about trust, privacy, and security in telesurgery.	AI algorithms (specific type not detailed) for training surgical robots	Blockchain with smart contracts and IPFS for secure, low-cost data storage	BITS improves TS by enhancing security and trust and reducing latency and storage costs, making remote surgery more feasible.	Current TS systems face unresolved security, latency, and storage cost challenges that need further refinement.
Jennath et al. [19]	2020	To address the current e-Health systems' privacy and security.	General mention of building AI models; specific algorithms not stated	Blockchain with immutable distributed data store for audit trail and consent provenance	Blockchain ensures secure, transparent data sharing and traceability for training trusted AI models in e-Health systems.	Current e-health systems lack security, privacy, and consent mechanisms, which this blockchain framework aims to address.
Kuo et al. [20]	2020	To offer a different approach to healthcare AI models that is built on decentralization.	EXpectation propagation logistic regression	Permissioned blockchain	ExplorerChain provides similar performance to centralized methods but decentralizes data coordination using blockchain, with a slight increase in running time.	It assumes institutions are honest-but-curious and has a trade-off in efficiency, especially with small datasets.
Kumar et al. [21]	2021	To guarantee privacy while permitting a flow of information to diagnose lung cancer using CT images.	RCNN, Bat algorithm	Not explicitly mentioned but involves a decentralized blockchain for distributing model weights	The proposed method improves lung cancer detection by securely sharing deep learning model weights via smart contracts and using data augmentation to handle different sizes of CT images.	Challenges in sharing medical data while maintaining privacy and potential computational complexity when dealing with large-scale data.
Tan et al. [22]	2021	To create an algorithm that manages a range of datasets from various countries and locations.	Deep learning algorithms for detecting myopic macular degeneration and high myopia	Blockchain-based AI platform for secure data transfer, model transfer, and model testing	The deep learning algorithms showed robust diagnostic performance, outperforming human experts in detecting myopic macular degeneration and high myopia. Blockchain technology was successfully used for data and model transfer across multiple sites and countries.	Challenges in AI medical studies include transparency, auditability, and traceability, which remain unresolved in this study.
Kumar et al. [23]	2021	To address concerns about data sharing between hospitals, including security and privacy, when building a model to identify COVID-19 patients from CT scans.	Capsule network-based segmentation and classification for detecting COVID-19 patients	Blockchain-based FL for authenticating data and training a global model while preserving privacy	The proposed framework demonstrates improved detection performance for COVID-19 patients using up-to-date data, with privacy-preserving FL and data normalization techniques.	Not reported
Nguyen et al. [24]	2021	To develop an intrusion detection system in the healthcare industry by utilizing blockchain-enabled transmission of data and classification algorithms.	DBN for intrusion detection and ResNet for disease classification	Blockchain technology for secure data transmission to the cloud server	The proposed model effectively performs data acquisition, intrusion detection, and disease classification while ensuring privacy and security through blockchain technology.	Not reported
Puri et al. [25]	2024	To address the data's non-transparency, security, privacy, and single-point failure problems in remote patient monitoring.	AI-enabled smart contracts for authentication and IoT node identification	Public blockchain network	The proposed framework enhances trust and transparency in healthcare records, improves energy consumption, reduces data request time, increases throughput, reduces latency, and lowers transaction fees.	Not reported
Khan et al. [26]	2021	To address the privacy of patient information and the time-consuming, expensive examination of anomalies in the stomach.	CNN with Softmax classifier, optimized using genetic algorithm and entropy function.	Blockchain-based approach with ledger blocks attached to each layer, the network, and cloud storage	The proposed method achieves 96.8% accuracy for stomach infection recognition and ensures system security by preventing tampering and modification attacks.	Not reported

Quality Assessment Results

The assessment revealed moderate-to-high methodological quality across studies (scores: 6-10/12). All studies explicitly stated objectives (score 2) and demonstrated innovation (score 2). However, reproducibility scored 0 universally due to missing datasets/code. High-performing studies (e.g., Kuo et al. [20] 10/12; Tan et al. [22] 9/12) excelled in methodology and evaluation but lacked reproducibility. Weaknesses included insufficient discussion of limitations (e.g., Kim & Huh [17], Nguyen et al. [24]) and vague technical descriptions (e.g., Jennath et al. [19]). Only 33% of studies rigorously addressed limitations, highlighting critical gaps in transparency and validity. These findings underscore the need for standardized reporting of technical details, open-source implementations, and deeper critical reflection in blockchain-AI healthcare research (Table 2).

**Table 2:** Quality Assessment of Studies Included Using JBI Critical Appraisal Checklist JBI, Joanna Briggs Institute

Study	Clear Objective	Methodology	Evaluation	Limitations	Reproducibility	Innovation	Total
Mamoshina et al. [12]	2	1	1	2	0	2	8
Połap et al. [13]	2	1	1	1	0	2	7
Zerka et al. [14]	2	2	2	1	0	2	9
Rahman et al. [15]	2	1	1	2	0	2	8
Pilozzi and Huang [16]	2	1	1	1	0	2	7
Kim and Huh [17]	2	1	1	0	0	2	6
Gupta et al. [18]	2	1	1	1	0	2	7
Jennath et al. [19]	2	1	1	1	0	2	7
Kuo et al. [20]	2	2	2	2	0	2	10
Kumar et al. [21]	2	1	2	1	0	2	8
Tan et al. [22]	2	2	2	1	0	2	9
Kumar et al. [23]	2	2	2	0	0	2	8
Nguyen et al. [24]	2	2	2	0	0	2	8
Puri et al. [25]	2	2	2	0	0	2	8
Khan et al. [26]	2	2	2	0	0	2	8

Discussion

This systematic review synthesized 15 studies to evaluate the role of blockchain in securing AI-driven healthcare systems. The findings reveal transformative potential but also expose critical gaps in technical rigor, scalability, and real-world applicability. Below, we interpret these results across five themes, compare them with existing literature, and propose pathways for advancing this interdisciplinary field.

Synergy Between Blockchain and AI

The integration of blockchain and AI addresses two core challenges in healthcare: data security and decentralized governance. Studies like Rahman et al. [15] and Zerka et al. [14] demonstrated how blockchain-managed FL frameworks could preserve privacy while enabling collaborative AI training across institutions. For instance, Zerka et al.’s [14] C-DistriM platform achieved performance parity with centralized models while ensuring transparency-a finding aligned with Khan et al. [4], who highlighted blockchain’s utility in mitigating data silos in multicentric research.

However, our review identified inconsistencies in blockchain architectures. While Kuo et al. [20] used permissioned blockchains to balance efficiency and control, Puri et al. [25] advocated for public blockchains to maximize transparency. This divergence mirrors debates in broader literature: Das et al. [10] argued that permissioned blockchains suit healthcare due to regulatory compliance, whereas public chains risk exposing sensitive data. Notably, none of the reviewed studies employed hybrid architectures (e.g., consortium blockchains), which could reconcile these trade-offs, a gap emphasized by Devarapu et al. [5] in their analysis of IoT healthcare systems.

The studies leveraged a spectrum of AI techniques, from convolutional neural networks [26] to NLP [16]. This aligns with trends in general AI healthcare research, where deep learning dominates diagnostic applications [6]. However, simpler models like logistic regression [20] were equally effective for specific tasks, suggesting that complexity does not always correlate with utility, a point underscored by Boi et al. [27] in their critique of AI overengineering.

Technical Innovations and Persistent Limitations

Several studies introduced novel frameworks for secure AI training. For example, Kumar et al. [21] combined blockchain with FL to share lung cancer detection models without exposing raw CT data, an approach validated by Deep et al. [28] in their work on distributed radiology workflows. Similarly, Nguyen et al. [24] used blockchain to secure intrusion detection systems, achieving 92% accuracy in disease classification. These innovations align with broader efforts to decentralize AI, such as Google’s Federated Learning of Cohorts (FLoC), though healthcare-specific solutions remain nascent.

Despite progress, critical limitations persist: None of the studies provided open-source code or datasets, hindering replication. This mirrors concerns raised by Ramachandran et al. [29], who found that <15% of AI healthcare studies share code, stifling progress. Kuo et al. [20] reported a 20% increase in runtime when using blockchain for decentralized coordination, a trade-off consistent with Himdi et al. [30], who noted blockchain’s inherent latency in large-scale systems. Many frameworks (e.g., Jennath et al. [19]) assumed "honest-but-curious" participants, ignoring malicious actors. This contrasts with healthcare-centric blockchain models like MedRec [31], which incorporated adversarial robustness.

Clinical and Operational Impact

Studies focusing on disease detection (e.g., Tan et al. [22]; Khan et al. [26]) demonstrated AI-blockchain systems outperforming human experts in tasks like retinal diagnostics and stomach anomaly recognition. These results echo Kasula’s [32] assertion that AI can reduce diagnostic errors, but blockchain’s role in ensuring data integrity remains underexplored. For instance, Tan et al. used blockchain for cross-border data transfer but did not quantify its impact on diagnostic accuracy, a missed opportunity to validate the synergy.

Gupta et al. [18] reduced telesurgery latency by 35% using blockchain-IPFS storage, while Puri et al. [25] lowered transaction fees by 40% in remote patient monitoring. These metrics align with operational benchmarks from non-healthcare blockchain applications (e.g., supply chain logistics by Saberi et al. [33]) but lack validation in real clinical settings. Notably, none of the studies addressed interoperability with legacy systems like EHRs, a barrier highlighted by McGhin et al. [34] as critical for adoption.

Ethical and Regulatory Challenges

Mamoshina et al. [12] proposed a patient-controlled health data marketplace, empowering individuals to monetize their data. While conceptually promising, this raises ethical concerns about commodification, a critique leveled by Mittelstadt [35] against commercial health platforms. Similarly, Jennath et al. [19] emphasized audit trails for consent management but overlooked dynamic consent models, which are gaining traction in General Data Protection Regulation (GDPR)-compliant systems [36].

Only two studies [20,25] mentioned compliance with regulations like the Health Insurance Portability and Accountability Act (HIPAA) or GDPR. This neglect contrasts with frameworks like Healthchain [37], which embedded regulatory checks into smart contracts. Without explicit alignment, blockchain-AI systems risk non-compliance, particularly in cross-border data transfers, a challenge underscored by the EU’s recent Data Governance Act.

Future Directions

A unified framework for blockchain-AI integration is urgently needed. Lessons could be drawn from the machine learning blockchain (MLBC) initiative, which standardizes model sharing via smart contracts. For healthcare, hybrid architectures combining permissioned blockchains (for compliance) and zk-SNARKs (for privacy) could balance efficiency and security.

Future studies must prioritize clinical trials. For example, deploying Kumar et al.’s [21] COVID-19 detection framework in multicenter trials could validate its scalability, a step absent in current research. Collaboration with healthcare providers, as seen in IBM’s Watson Health partnerships, is critical.

Incorporating ethics-by-design principles, such as the EU’s Ethics Guidelines for Trustworthy AI, can mitigate risks like bias. For instance, Khan et al.’s [26] stomach anomaly detector achieved 96.8% accuracy but did not audit for demographic bias, an oversight critiqued by Obermeyer et al. [38] in similar models.

Advancing this field requires collaboration across computer science, healthcare, and policy. Initiatives like the IEEE Standards Association’s working group on blockchain in healthcare could provide governance blueprints, while funding bodies like NIH should prioritize grants for translational research.

## Conclusions

This review underscores the transformative potential of blockchain-AI integration in healthcare, particularly in enhancing security, transparency, and diagnostic precision. However, the field remains nascent, with studies often prioritizing theoretical novelty over practical validation. By addressing gaps in reproducibility, scalability, and ethical governance, researchers can transition from proof-of-concept frameworks to real-world solutions. As healthcare grapples with escalating data breaches and AI adoption, blockchain’s role as a trust anchor will be pivotal, but only if the community embraces rigor, collaboration, and patient-centric design.
